# Dysbiosis of Oral Microbiome: A Key Player in Oral Carcinogenesis? A Critical Review

**DOI:** 10.3390/biomedicines13020448

**Published:** 2025-02-12

**Authors:** K. Devaraja, Sadhna Aggarwal

**Affiliations:** 1 Department of Head and Neck Surgery, Kasturba Medical College, Manipal, Manipal Academy of Higher Education, Manipal 576104, India; 2 Department of Radiation Oncology, The University of Texas MD Anderson Cancer Center, Houston, TX 77030, USA

**Keywords:** microbiome, microbiota, dysbiosis, oral cancer, next-generation sequencing, Fusobacterium, *Porphyromonas*, *Candida*, human papilloma virus, acetaldehyde

## Abstract

The oral cavity is known to harbor hundreds of microorganisms, belonging to various genera, constituting a peculiar flora called the oral microbiome. The change in the relative distribution of the constituents of this microbial flora, due to any reason, leads to oral dysbiosis. For centuries, oral dysbiosis has been linked to the etiopathogenesis of several medical illnesses, both locally and systemically-. However, aided by the recent advent of bio-technological capabilities, several reports have re-emerged that link oral dysbiosis to oral carcinogenesis, and numerous studies are currently exploring their association and plausible mechanisms. Some of the proposed mechanisms of oral dysbiosis-induced carcinogenesis (ODIC) include—a bacteria-induced chronic inflammatory state leading to direct cellular damage, inflammatory-cytokine-mediated promotion of cellular proliferation and invasion, release of bacterial products that are carcinogenic, and suppression of local immunity by alteration of the tumor microenvironment. However, the actual interactions between these cellular mechanisms and their role in carcinogenesis are not yet fully understood. This review provides a comprehensive overview of the various hypotheses and mechanisms implicated in the ODIC, along with the corresponding molecular aberrations. Apart from discussing the usual constituents of the oral microbiome profile, the review also summarizes the various dysbiosis profiles implicated in ODIC. The review also sheds light on the potential clinical implications of the research on oral microbiome in the prevention and management of oral cancer.

## 1. Introduction

Even though the first microorganism was observed and characterized by Robert Hooke and Antoni van Leeuwenhoek three and half centuries ago, the relevance of microbes in human health and disease, particularly their association with non-communicable diseases, has been realized only at the turn of the last century [1,2]. Owing to the recent biotechnological advent, the identification and characterization of various microorganisms have become feasible and reproducible, allowing researchers to explore the role of these microorganisms in etiopathology and therapeutic implications of clinical conditions, including neoplastic diseases.

Oral cancer is one such clinical condition whose pathophysiology has been linked to altered microbial flora in the oral cavity. While the most prevalent risk factor for the oral carcinogenesis continues to be the exposure to tobacco and alcohol, particularly in developing countries, a proportion of the affected patients tend to have no exposure to any of the known risk factors [3]. The oral microbiota has been attributed as one of the possible etiological factors in such cases [4]. However, in the absence of direct evidence on their etiopathological role, there is no consensus on how to interpret the currently available date on associations between some of the microbiological agents and oral cancer. In other words, despite the numerous pre-clinical and clinical studies published over the decades, there is no agreement among the scientists on the ‘causal link’ between the oral microbiome and oral carcinogenesis, and no pharmacotherapeutic has been approved to date for use in oral cancer that targets microbiota [5,6]. Since oral cancer continues to be one of the most common cancers globally, with no change in its therapeutic outcomes over the last few decades, there is an urgent need to evaluate the relationship between the oral microbiome and oral cancer for its potential therapeutic implications, as well as for developing preventive strategies [7,8].

## 2. Fundamentals of the Microbiome

Microbes have been integral components throughout the evolution of life on earth and continue to play a crucial role in the health and disease of most living organisms. However, the science around the study of microorganisms, particularly in medicine, has garnered attention only in the last few centuries. Since the advent of next-generation sequencing and metagenomic analysis capabilities, the field of the microbiome has advanced in leaps and bounds, culminating in the conceptualization of the human microbiome project (HMP) in 2007, with the participation of several centers around the world [9]. The HMP has contributed substantially to our understanding of microbiomes and their various clinical implications. The second phase of HMP, called integrative HMP, was completed in 2019, covering dynamic changes in the microbiome and host under three clinical conditions [10]. 

### 2.1. Origin of the Term ‘Microbiome’

The word ‘micro’ is of Greek origin, meaning small, and the term ‘biome’ is derived from the Greek word ‘bíos,’ meaning life. The word ‘biota’, which is also from ancient Greek, means the living organisms of an ecosystem or a particular area [11]. Interestingly, the credit for coining the term ‘microbiome’ has been bestowed to Joshua Lederberg, an American molecular biologist who received half of the 1958 Nobel Prize in Medicine at the age of 33 for his work on microbial genetic recombination [12,13,14,15,16,17]. However, even though most the scientific reports to date cite Lederberg’s work from 2001 for “the origin of the term microbiome”, Lederberg does not mention the term in that report [15,16,17,18,19,20]. The relevance of microbes and their interactions with numerous bodily functions had already been discussed by several reports published prior to 2001, some of which had even used terms such as ‘microbiome’ and ‘microbiota’ in their descriptions [21,22,23,24]. Interestingly, John M. Whipps, a professor from the University of Warwick (UK), and his colleagues, Karen Lewis and Roderic C. Cooke, used the term microbiome in one of their textbooks published in 1988, and provided a definition, which stands as valid till date [21,23]. They considered the microbiome as a “convenient ecological framework in which to examine biocontrol systems” and defined it as the “characteristic microbial community occupying a reasonably well-defined habitat which has distinct physico-chemical properties”. “The term thus not only refers to the microorganisms involved but also encompasses their theatres of activity [21,22,23]”. Even though several reports over the years have used the terms ‘microbiome’ and ‘microbiota’ as synonyms and interchangeably, today there is a consensus among researchers about the demarcation between these terms [11]. The term ‘microbiota’ typically applies to the assemblage of living microorganisms, such as bacteria, fungi, archaea, other eukaryotes, and viruses, present in a defined environment, and the term ‘microbiome’ represents the entire habitat, including the microorganisms, their genomes, and the surrounding environmental conditions [15,16,25]. In simple words, the “microbiome is a biological system of heterogenous communities of microorganisms living in the same habitat, engaging in non-linear and dynamic interactions” [26].

### 2.2. Human Microbiome, an Independent Organ System 

Although it has not been possible to ascertain the exact number of microbes associated with humans, it is estimated that a human body houses several trillions of microbes, belonging to hundreds of species, distributed across various body areas [9,27,28,29]. Most of the concentration of the microbiota in humans is seen in the skin, oral cavity, gut, respiratory apparatus, and genitourinary organs [27,30]. The gut microbiota, being the largest, is also considered the most significant for maintaining human health, followed by the oral microbiota, which is regarded as the second largest microbial community in humans [27,29,30]. These microbes interact with each other and with the host, and these ecological interactive patterns can be classified as positive (mutualism, synergism, or commensalism), negative (predation, parasitism, antagonism, or competition), or neutral (where there is no effect on the functional capacities or fitness of interacting species) [11]. While most of the microbiota in humans are commensal in nature, several symbiotic as well as pathogenic microorganisms are also known to co-exist in the same region. There exists a significant variation between humans in terms of the composition and functions of the microbiome, as each individual tends to harbor diverse microbial communities at different body sites [27]. Accordingly, the human microbiome can be broadly classified into two types. A ‘core microbiome’ is nothing but a shared microbiome found in a given habitat (e.g., gut, mouth, skin) in all individuals and is comprised of the predominant species that exist under healthy conditions at different sites of the body. The other is a ‘variable microbiome’ that is exclusive to the individual and has evolved over time in response to a combination of factors—unique lifestyle, environmental exposures, and phenotypic and genotypic determinants [9,31]. Nevertheless, each of these microbiomes, being considered as part of an independent organ system by itself, actively participates in regulating local homeostasis and several crucial bodily functions of the host [25,29,30,32]. In humans, the microbiomes of various sites play a vital role in imparting immunity, both locally as well as systemically, aid digestion and metabolism of the ingested food, and contribute to the synthesis of vitamins and other nutrients [27,29]. Since microbial composition influences the functioning of the ecosystem, it is important to understand and measure the diversity of human microbiota, and for this measurement of diversity, the species is considered the fundamental unit of analysis [33,34,35]. Diversity within a given community of microbes is referred to as alpha (α) diversity and is usually described using—the total number of species (species richness), the relative abundances of the species (species evenness), or indices that combine these two dimensions. Similarly, the measurement of diversity among communities or along an environmental gradient constitutes beta (β) diversity, which is often characterized using the number of species shared between the two communities. The gamma (*γ*) diversity is measured as the total observed richness of all samples within a habitat. In simple terms, the number of species in a single sampling unit reflects *α* diversity, the variability in species composition (dissimilarity or similarity) among communities represents β diversity, and the overall number of species within a defined geographical area or habitat constitutes *γ* diversity [33,34,35]. Lastly, the term ‘dysbiosis’ represents any disruption in the natural microbiome profile with respect to a relative abundance of the constituent microbes of a particular anatomical space, which affects the functional ability of that microbiome. Dysbiosis is different from diversities in that it reflects not just the alteration in microbiota but also the consequent alteration in the biological activity of the microbiome. While the diversities measure the variations in microbes, dysbiosis represents the variation in the microbiome, and the latter is implicated in etiopathology of various clinical conditions, both infectious and non-communicable diseases, including cardiovascular disorders, autoimmune conditions, and cancers [27,29]. Generally, the microbial profiles of different areas of the body could differ considerably, but the profiles of the microbiota found in various sites, such as the oral cavity and gut, are specific to the host, with a strong temporal stability. The human digestive tract tends to harbor a large number of microbes but exhibits a limited diversity at the phylum level compared to that of the oral cavity.

### 2.3. Oral Microbiome

The oral microbiome is known for its most versatile microbiota, which is predominated by bacteria of 500–1000 species, followed by a varieties of fungi, viruses, protozoa, and archaea [27,31,36,37]. 

#### 2.3.1. Bacteria in the Oral Microbiome

Studies have shown that around 80–96% of the taxa in oral microbiota belong to the six major phyla of bacteria, Firmicutes (genus *Streptococcus*, *Gemella*, *Eubacterium*, *Selenomonas*, *Abiotrophia*, *Granulicatella*, *Selenomonas*, *Veillonella*), Bacteroidetes (genus *Prevotella*, *Capnocytophaga*, *Porphyromonas*, *Bacteroides*, *Tannerella*, *Bergeyella*), Proteobacteria (genus *Neisseria*, *Eikenella*, *Campylobacter*, *Haemophilus*), Actinobacteria (genus *Corynebacterium*, *Rothia*, *Actinomyces*, *Microbacterium*, *Propionibacterium*, *Mycobacterium*), Spirochaetes (genus *Treponema*), and Fusobacteria (genus *Fusobacterium* and *Leptotrichia*) [36,38,39,40,41,42,43,44]. The other phyla are Euryarchaeota, Gracilibacteria (GN02), Chlamydiae, Chloroflexi, SR1, Synergistetes, Tenericutes, and Saccharibacteria (TM7), which account for around 4% of the oral microbiota [36,45]. Tenericutes (*Mycoplasma* species) is a recently created phylum, belonging to class *Mollicutes*, which was previously known to be within the Firmicutes [36]. At the genus level, *Streptococci* has been identified as the most abundant genus in mucosal sites, followed by *Neisseria*, *Prevotella*, and *Haemophilus* genera, and in subgingival plaque specimens, mostly are represented by anaerobes (*Actinomyces*, *Veillonella*, *Fusobacterium*) [40]. At the species level, *Streptococcus mitis* is the most prevalent organism to have been identified in the oral microbiome, followed by *Granulicatella adiacens* and *Veillonella* spp., detected at almost all sites [40,42]. *Streptococcus oralis*, S. *salivarius*, and S. *sanguinis*, *Haemophilus parainfluenzae*, *Prevotella melaninogenica*, *Neisseria subflava*, and *Rothia dentocariosa* are the other prevalent species identified of the respective genera [40,41,42]. The *Mycoplasma* species (most commonly *M. salivarium*, followed by *M. orale*) has been noted in 97% of the saliva [46]. Nevertheless, according to various reports, up to around 60% of the bacterial flora from the human oral microbiome is represented by not-yet-cultivated phylotypes [18,36,42,45,47]. 

#### 2.3.2. Other Components of Oral Microbiota 

On the other hand, fungi and viruses are less abundant in the oral microbiome, accounting only for less than 0.1% of the total microbial counts [40]. Among the mycetes, the *Candida* genus, particularly *C. albicans* species, is the most prevalent fungus found in oral microbiota, followed by *Cladosporium*, *Aureobasidium*, *Saccharomycetales*, *Aspergillus*, *Fusarium*, and *Cryptococcus* [48]. Although the distribution of various fungal species in oral cavity differs across the studies, *Candida* is the most frequently isolated genus, found in 65–75% of healthy individuals, and thus, can be considered a ‘core mycobiome’ [44,48,49]. With regards to the oral virome, eukaryotic viruses and phages predominate [37,40]. Eukaryotic viruses, which mainly include *Anelloviridae*, *Herpesviridae*, and *Papillomaviridae*, and bacteriophages belonging to the *Caudovirales* order (*Siphoviridae*, *Myoviridae*, and *Podoviridae* families) are commonly detected in oral microbiota [37,40,45,50]. Lastly, the oral microbiota also consists of protozoa, among which *Entamoeba gingivalis* and *Trichomonas tenax* are the most commonly found, and these are mainly saprophytic [45]. The detailed description of the human-associated oral microbiota, including their phylogenetic and taxonomic structures and the scheme of classification, has been curated into a phylogeny-based database called the Human Oral Microbiome Database (HOMD), which can be accessed at https://www.homd.org/ [36,51]. According to the HOMD, around 772 prokaryotic species have been identified in oral microbiota so far, belonging to 185 genera, of which only 54% are officially named [45,51].

### 2.4. Physiological Determinants of Oral Microbiome

#### 2.4.1. Source of Oral Microbiota and Variations with Age

The oral microbiota, even though is already established at the time of birth, is shaped dynamically over the next few years of life, depending on several physical, environmental and social factors [43,52,53,54,55,56]. From a few bacterial agents at birth, the numbers and diversity of the microbiota inside the oral cavity increases over the first few years of childhood [43,52,53,54,55,56]. At first, newborns acquire microbiota vertically from the mother, depending on the mode of delivery and the pattern of breastfeeding [54,55]. The key components of oral microbiota in infants are *Streptococcus salivarius* and *Lactobacillus* spp. [57]. Subsequently, the oral microbiome changes in infancy, due to the horizontal transmission of bacteria from the other family members who live in the same environment, via eating and drinking habits, type of food consumed, and personal hygiene practices [54,55,57]. This transition is predominantly marked by the increasing proportion of anaerobic species [58]. As per the literature, the foundation of adult types of oral microbiota is said to be established by the age of 18 months to five years, while there could be variations in the profile in some children due to age-related environmental influences [52,53,56,58]. 

#### 2.4.2. Intraoral Environment

The oral cavity has been identified as having several distinct habitats for microbial communities, such as the buccal mucosa, gingiva, hard palate, tongue, tonsils, subgingival/supragingival plaque, and saliva [30,59,60]. The distribution of the above-mentioned microbial species inside the oral cavity and crevicular fluid exhibits a strong site-tropism, as influenced by various physical and chemical factors, such as surface topology, epithelial tissue structure, and oxygen availability [32,61]. Generally, the oxygen-rich oral tissue surfaces and saliva support aerobic species and exhibit Firmicutes as a predominate phylum [30,32,59]. Contrastingly, oxygen-deprived subgingival surfaces predominantly harbor anaerobic bacteria and are dominated by Firmicutes, Bacteriodetes, Actinobacteria, Proteobacteria, and Fusobacteria [30,32,59]. Furthermore, the presence of tissue crypts, taste buds, and the ability to retain bacterial metabolites in their local environments also influence the composition of microbiota in different sub-sites inside the oral cavity [62]. In other words, due to the differential environmental conditions, each of the specific sub-sites of the oral cavity tends to harbor its own microbiota, with a distinct distribution at the phylum level. Interestingly, the emerging evidence has also identified the role of interspecies interactions in determining the profile of microbiota and, thus, the function of the whole microbiome [62].

### 2.5. Oral Dysbiosis

Oral dysbiosis, in which the relative abundance of microbiota of a particular site or organ changes significantly to alter the physiological functions of the microbiome, has been shown to play a crucial role in the pathophysiology of several clinical conditions, both locally and systemically. The local conditions that have shown an association with oral dysbiosis include dental caries, periodontitis, oral potentially malignant disorder (OPMD), jaw tumors, and invasive oral cancer, most of which are attributed to local pro-inflammatory changes inside the oral cavity caused by dysbiotic bacteria [38,63,64,65,66]. Similarly, the oral dysbiosis-mediated immune changes are implicated in the etiopathogenesis of several systemic diseases, affecting cardiovascular, respiratory, endocrine, nervous, and gastrointestinal systems [29,30,32,67,68,69,70,71,72,73,74]. Studies have also shown a significant association between oral dysbiosis and benign and malignant neoplasms, particularly those arising from the gastrointestinal tract and the oral cavity [30,75,76,77]. Although there are a few controversial views on these associations, emerging evidence in recent times link oral dysbiosis to the etiopathology of many of these conditions. For the obvious reason of anatomical proximity, the association of oral dysbiosis with oral cancer has found major attention in this regard by garnering significant supportive evidence. As per a recent bibliometric analysis of scientific works that have evaluated ‘the role of the oral microbiome in oral cancer’ over the last 10 years, there has been a drastic increase in global interest on this topic, and most of the leading publications in the last decade have come from China and the United States, followed by India, Finland, Japan, and Taiwan [78].

## 3. Oral Dysbiosis in Oral Carcinogenesis

The oral microbiome in humans consists of over a hundred different taxa or genera and several hundreds of species-level phylotypes, and traditional microbial culture and Sanger sequencing for their identification is not practical, affordable, analyzable, and reproducible [45]. The surge in the recent studies on oral microbiome and the identification of the various profiles of dysbiosis has been made possible by the recent biotechnological advent, with the introduction of innovative sequencing and amplifying technologies, and the curation of HOMD based on the 16S rRNA sequencing, which together have enabled researchers to look beyond conventional culture techniques for identifying and characterizing the components of oral microbiota in health and disease states [79,80,81,82,83]. 

### 3.1. Profile of Oral Microbiota in Oral Cancer

As mentioned earlier, of the numerous phyla of microbes, four to six phyla account for most of the oral microbiota, both in a healthy state and in a dysbiosis state [47,84,85,86,87,88,89,90,91]. Although some variations exist between the studies in terms of the identified relative abundance of various microbiota in their patients, broadly, the reduced abundance of Firmicutes and Actinobacteria, along with a significant enrichment of Spirochaetes, Fusobacteria, and Bacteroidetes, is the most appreciated dysbiosis profile in oral cancer patients [91,92,93]. In several clinical studies, as against the anatomically matched contralateral clinically normal tissue sample or samples from healthy controls, the microbiota from oral cancer tissue has demonstrated higher abundance of genus *Fusobacterium*, *Prevotella*, *Alloprevotella*, and *Porphyromonas* with significant reductions in the *Streptococcus*, *Veillonella*, and *Rothia*, [84,87,88,89,94,95,96,97]. Studies have also shown higher concentrations of *Peptostreptococcus, Treponema*, *Parvimonas*, *Capnocytophaga*, and *Leptotrichia* in tumors [88,97]. In one study, apart from lower abundance of commensals such as *Corynebacterium*, *Actinomyces,* and *Haemophilus*, bacteria belonging to genera *Selenomonas* and *Mycoplasma* were overexpressed more in oral cancer patients than in healthy controls [98]. Nevertheless, at the species level, the periodontal pathogens, namely, *F. nucleatum*, *Porphyromonas gingivalis*, and *Treponema denticola*, are the most commonly identified species in oral cancer, followed by *Porphyromonas endodontalis*, *Prevotella tannerae*, *Prevotella intermedia*, *Pseudomonas aeruginosa*, and *Campylobacter* sp., along with the reduction in *S. mitis*, *Rothia mucilaginosa*, *Haemophilus parainfluenzae*, and *Veilonella parvula* [84,85,87,88,92,94,96,99]. The latest studies have also identified an association between the head and neck cancer, including the oral cancer, and a few novel bacterial species, such as *Prevotella salivae*, *S. sanguinis*, *Streptococcus anginosus*, *Leptotrichia* species, and *Peptostreptococcus anaerobius* [82,86]. Studies have also demonstrated a progressive enrichment of the periodontal pathogens from normal healthy controls through OPMD to invasive oral cancer, paralleling the sequential reduction of commensal *Streptococcus* in the microbiome [95]. In one study, the concentration of *F. periodonticum* and *Parvimonas micra* also increased with the progression of oral cancer from stage I to stage IV, while that of *S. mitis and Porphyromonas pasteri* reduced significantly [89]. 

### 3.2. Mechanisms of Oral Dysbiosis-Induced Oral Carcinogenesis 

Oral dysbiosis can be involved in the process of carcinogenesis majorly by two major approaches: by producing pro-carcinogenic metabolites or substances and through immune mechanisms that induce or facilitate proliferation or that hinder apoptosis [76,100]. Furthermore, the various effector mechanisms identified at the cellular level include upregulation of cell survival factors, release of mutagenic substances that induce of epithelial-to-mesenchymal transition (EMT) and trigger synthesis of inflammatory cytokines or growth factors, impairment of anti-tumor immunity, and facilitation of tumor angiogenesis [83,101]. There are numerous other in vivo reports on the role of *P. gingivalis*, *F. nucleatum*, and a few other oncobacteria in either inducing or facilitating the proliferation of oral cancer, as discussed below. 

#### 3.2.1. Molecular Mechanisms

In 2015, Binder Gallimidi et al., from Israel, showed an etiological role of periodontal pathogens in oral cancer by establishing a murine model of chronic infection-associated oral tumorigenesis [102]. In this model, they showed that *P. gingivalis* and *F. nucleatum*, two well-known periodontal pathogens, can directly stimulate cancerous cells and promote proliferation through various promoting factors such as TLR2, NF-κB signaling, IL-6, and TNFα. The authors summarize their results by inferring, “exposure of oral epithelial cells to *P. gingivalis/F. nucleatum* triggers TLR signaling, resulting in IL-6 production that activates STAT3, which in turn induces important effectors that drive the growth and invasiveness of the oral cavity squamous cell carcinoma [102]”. Several other independent studies also support these results. For instance, *P. gingivalis* promoted cell invasion and proliferation in an oral cancer mouse model via JAK1/STAT3 signaling pathway activation and EMT in tumor microenvironment (TME) [103]. In a cell proliferation model, *P. gingivalis* promoted oral cancer cell proliferation by regulating cyclin D1 expression via the miR-21/PDCD4/AP-1 negative feedback signaling pathway [104]. Another study has shown that *P. gingivalis* can induce the noncanonical activation of β-catenin, a major pathway in the control of cell proliferation and tumorigenesis [105]. Furthermore, in oral cancer cell culture and animal models, *P. gingivalis* suppressed antigen-specific CD8+ T cells through upregulation of PD-L1 expression on dendritic cell (DC)s by increasing the phosphorylation of Akt and STAT3 [106]. On the other hand, though several in vivo studies have highlighted the anti-apoptotic mechanisms of *P. gingivalis* in epithelial cells, their specific role in cancer progression seems to have not yet been studied [107,108,109,110]. 

Studies have shown the activation of multiple pro-carcinogenic pathways after the in vivo infection of oral cancer cell lines with *F. nucleatum*. *F. nucleatum* has been shown to inhibit the activities of natural killer (NK) cells and cytolytic T cells by two tumor-immune evasion mechanisms—by interacting with ‘T cell immunoglobulin and ITIM domain’ (TIGIT) expressed on all NK cells and by activating CEACAM1, a member of the carcinoembryonic antigen-related cell adhesion molecules (CEACAMs), which serves as an inhibitory receptor on various immune cells [111,112,113]. Activation of CEACAM1 impairs the normal maturation of immature DCs, suppresses lymphocyte responses to activating stimuli, causes T cell exhaustion, and also impairs phagocytic engulfment of the bacteria [112,113]. In another study, *F. nucleatum* upregulated MMP1, MMP9, and IL-8 in oral cancer cell lines, and the infected cell lines showed overexpression of cell survival markers, such as MYC, JAK1 and STAT3, and EMT markers (ZEB1 and TGF-β) [114]. In this study, the Fusobacterial culture supernatant (primarily Lipopolysaccharide) was sufficient to induce IL-8 secretion, suggesting that “direct contact of live Fusobacteria with cancer cells might not be required to exert changes in cancer cell behavior [114]”. In another study, *F. nucleatum*-infected tongue squamous carcinoma cells exhibited increased proliferation ability due to an accelerated cell cycle, with a reduced expression of p27 and downregulated Ku70 and wild p53 [115]. In a xenograft tumor mode, it was shown that intratumoral *F. nucleatum*, enriched inside invasive oral cancer tissue, can drive the formation of tumor-associated macrophages, which facilitate immune escape [116]. This action was mediated by the bacteria-triggered GalNAc-Autophagy-TBC1D5 signaling, leading to GLUT1 aggregation in the plasma membrane and the deposition of extracellular lactate [116]. Furthermore, Li et al. have shown that *F. nucleatum* promotes the proliferation of oral cancer cell lines through the CDH1/β-catenin pathway, G2/M phase arrest, activating cyclinD1 and Myc [117]. These in vitro findings were also supported by animal models of the oral tumors, in which the mice infected with *F. nucleatum* developed significantly larger and more numerous lesions compared to those not infected and also demonstrated a higher Ki-67 proliferation index [114,117]. 

In an in vivo study, all three key periodontal pathogens, *F. nucleatum*, *P. gingivalis*, and *T. denticola*, independently enhanced oral cancer cell migration, invasion, tumorsphere formation, and tumorigenesis via crosstalk between TLR/MyD88 and integrin alpha V and FAK activation [118]. *T. denticola* has been shown to promote the growth of oral tumors in mice, along with upregulation of Ki67 expression and intracellular TGF-β pathway activation [119]. Interestingly, according to one of the reports, *Staphylococcus aureus*, a pathogen prevalent in oral cancer, can also induce the infection-associated malignant transformation of the oral epithelium through the activation of the COX-2/PGE_2_ pathway [120]. There are a few reports that have also suggested the role of *S. anginosus*, *V. parvula*, *P. endodontalis*, and *P. anaerobius* in the development of oral cancer [83,86]. 

#### 3.2.2. Metabolites as an Intermediate Between Bacteria and Carcinogenesis 

Several studies of the functional prediction of oral dysbiosis have shown that the oncogenic bacteria could also alter the TME by upregulating metabolites that are required to support cellular proliferation, attenuate anti-cancer effects, and prevent apoptosis [94,99,121]. In a study by Su et al., as compared to the contralateral normal tissue, the buccal mucosa cancers, apart from demonstrating an enrichment of genus *Fusobacterium* (species *F. nucleatum*) and a loss of genus *Streptococcus* (species *S. pneumoniae*) in the tumor sites, also showed decreased production of tumor-suppressive metabolites at the TME [94]. In a community-wide metatranscriptome analysis, Fusobacteria, which was rich in oral cancer patients over healthy controls, was associated with higher metabolic activities, such as iron ion transport, tryptophanase activity, peptidase activities, and superoxide dismutase, along with the upregulated activities related to capsule biosynthesis, flagellum synthesis, assembly, and chemotaxis [121]. In a study by Al-Hebshi, tumor sites enriched with *F. nucleatum* also showed upregulation of genes involved in bacterial mobility, flagellar assembly, bacterial chemotaxis, and lipopolysaccharid synthesis, as compared to the normal controls, who exhibited genes responsible for deoxyribonuclease (DNA) repair and other protective pathways [99]. Interestingly, in a nested case-control study, although the microbiome composition was not associated with an increased risk of head and neck cancer, a greater abundance of the commensal bacterial genera *Corynebacterium* and *Kingella* were protective against carcinogenesis, which could be attributed to their capacity to metabolize carcinogens [122]. 

Apart from bacterial colonies, several viruses, such as Human papillomavirus (HPV), Human immunodeficiency virus (HIV), and Epstein Barr virus (EBV), and a few fungal agents, particularly virulent strains of *Candida* have also been linked to oral carcinogenesis. While viral etiology for oral and oropharyngeal cancer, particularly related to HPV, is a major topic of discussion and goes beyond the scope of this review on the microbiome, interested readers can refer to our previous review for further details [123]. On the other hand, the etiopathological role of fungal agents corresponds to that of the bacteria and thus is elaboratedbelow. The *Candida* species, which is known to colonize OPMDs, including oral leukoplakia and oral lichen planus, is speculated to play a role in the transformation of OPMD into an invasive cancer [124]. It has been shown that the *Candida* isolates from the oral cancer lesions exhibited a significantly higher attribute of virulence than the isolates from chronic candidiasis and asymptomatic carriers, suggesting the role of *Candida* in the pathogenicity of oral cancer [125]. Nevertheless, studies have also shown a notable association of oral cancer with the enhanced virulence attributes of *Candida* yeasts, such as higher capacity to form biofilms and produce phospholipase and proteinase enzymes, and its ethanol-derived acetaldehyde production capability [125,126]. Of these, the production of acetaldehyde seems to have considerable contribution in the process of carcinogenesis. 

#### 3.2.3. Acetaldehyde in Dysbiosis-Driven Oral Carcinogenesis

Acetaldehyde, a component of alcoholic beverages, is a group 1 carcinogen, which reacts with DNA, producing DNA damage, chromosomal aberrations and DNA adducts that may, in turn, lead to mutations [127,128]. Although alcohol-derived acetaldehyde is generally metabolized in the liver, acetaldehyde can also be derived from alcohol in the oral cavity, courtesy of the peculiar oral microbiome [129]. In fact, the oral microbiome, including the *Candida*, increases the acetaldehyde concentrations in the oral cavity by more than one mechanism.

First, some components of oral microbiota have an intrinsic enzymatic property that mimics alcohol dehydrogenase (ADH), an enzyme that produces acetaldehyde from alcohol. Studies have shown that apart from *Candida* yeasts, bacteria belonging to *Neisseria*, *Streptococci*, and *Prevotella*, also show ADH-like enzymatic activity [130,131,132,133]. As a result, these microbes increase the conversion of alcohol to acetaldehyde in the oral cavity, in addition to that being produced by the natural ADH present in the oral mucosa [134,135]. Several *Candida* species, including *C. albicans* and non-*C. albicans* sp., have been shown to produce significant and mutagenic amounts of acetaldehyde in the presence of ethanol and glucose [136,137]. Interestingly, in one of these studies, the production of acetaldehyde was higher among smokers compared to non-smokers [136]. This could be because the tobacco smoke, apart from itself containing acetaldehyde, can also alter the oral microbiome to facilitate the production of a larger amount of acetaldehyde from ingested alcohol [134,138]. This also explains, at least partly, the plausible role of the oral microbiome in mediating the synergistic effect of tobacco and alcohol in oral carcinogenesis. In other words, while the alcohol and tobacco smoking can independently as well as cumulatively affect the oral microbiome, the resultant dysbiosis could contribute to the etiopathogenesis of oral cancer by potentiating the mutagenic activity of alcohol and tobacco [138,139]. 

Second, oral dysbiosis can produce acetaldehyde independent of alcohol ingestion, by a less-known clinical condition called auto-brewery syndrome (ABS) [129,134]. ABS, also known as ‘endogenous ethanol fermentation syndrome’, is seen in some individuals, wherein the affected person manifests endogenous production of alcohol, mediated by a super acetaldehyde-producing microbiome [140,141,142,143,144]. This phenomenon of ABS is attributed to the ‘crabtree effect’, and apart from the oral cavity, it is also known to affect the gut and urinary bladder [140,142,145,146]. Here, the ingested carbohydrate first undergo glycolysis to produce pyruvate, which will be followed by the decarboxylation of pyruvate into acetaldehyde, as catalyzed by pyruvate decarboxylase [145]. Generally, some of this acetaldehyde is further metabolized into ethanol by ADH present in the oral microbiome and in oral mucosa, while most of it is converted into a non-harmful molecule ‘acetate’ by the action of the acetaldehyde dehydrogenase (ALDH) present in the oral mucosa. However, in some individuals, owing to genetic polymorphisms of ALDH, the ALDH in oral mucosa may not be active, leading to the rise of mutagenic concentrations of acetaldehyde in the saliva [129,134]. Since the innate susceptibility to metabolize acetaldehyde can vary from person to person, the impact of ABS on oral carcinogenesis has not yet been accurately quantified. Although normal individuals could also manifest the symptoms and the effects of ABS, it is more commonly seen in patients with co-morbidities such as diabetes mellitus, obesity, non-alcoholic fatty liver disease, cirrhosis, hepatitis, Crohn’s disease, short bowel syndrome, conditions after resection of a part of the intestine, chronic intestinal pseudo-obstruction, and small intestinal bacterial overgrowth syndrome [145]. Nevertheless, these ABS-affected patients can independently produce acetaldehyde from various other sources, including ordinary food and beverages that do not contain alcohol. As a result, this phenomenon of ABS can be held responsible for the development of oral cancer in some of the patients who do not consume tobacco or alcohol, and thus, the oral dysbiosis could make a case as an ‘independent risk factor’ for oral carcinogenesis [4,134,147].

### 3.3. As a Biomarker and Therapeutic Significance

As per the literature, the oral microbial profile can be used as a marker for screening, diagnosing, and even prognosticating oral cancer, although there are several lacunae in these approaches [87,88,89,90,94,95,148]. Studies have shown that a typical profile of oral dysbiosis, in the form of enriched *F. nucleatum* and a decrease in *S. pneumoniae*, can be a reliable marker in predicting oral cancer [87,94]. There is a proposal by Ganly et al. to classify the oral microbiome into two types, periodontal pathogen–low (PPL) and periodontal pathogen–high (PPH) types, which reflect the relative abundance of three periodontal oncobacteria, including *Prevotella*, *Fusobacterium*, and *Alloprevotell* on one side and the higher abundant *Streptococcus* on the other [95]. They found this classification to be statistically reliable in its ability to separate the oral cancer lesions from normal tissue [95]. Interestingly, in one of the studies, a bacterial panel, that consisted of an increased *F. periodonticum* and *P. micra*, as well as reduced *S. mitis and P. pasteri*, exhibited a strong discriminating ability to separate stage IV oral cancer from the healthy controls [89]. Another study suggests an abundance of *Lactobacillus* or a loss of *Haemophilus, Neisseria, Gemellaceae*, or *Aggregatibacter* in saliva as a biomarker for head and neck cancer [90]. Further, microbiota composition in saliva has also been suggested for its potential use as a biomarker for predicting the pathologic transition of oral epithelial precursor lesions to cancer [148]. However, these sorts of approaches are barely useful for clinical applications in oral cancer, as most of the lesions of oral cancer are clinically appreciable, and the diagnosis mandates pathological confirmation to rule out closely associated OPMDs, which warrant a different therapeutic approach. In addition, the microbiota of oral cancer could be similar to that found in the OPMD lesions [84]. Furthermore, the oral microbial profile, as a non-invasive test (liquid biopsy), lacks the sensitivity and specificity to diagnose oral cancer and might only work as an adjuvant in cases with deep-seated lesions. 

With regard to the prognostic ability of oral microbiota, the literature is marred with contradicting reports. While many studies have reported poor survival with *F. nucleatum* positivity in oral cancer, Neuzillet and colleagues have found oral cancers with *F. nucleatum* to be associated with a favorable prognosis [149]. Similarly, there are reports supporting the association of *Fusobacterium* in patients without lymph node metastasis over those with lymph node metastasis, as well as vice versa [89,100]. In one study, *Schlegelella* and *Methyloversatilis* were associated with poor prognosis in head and neck cancers, and a richer tumor microbiota with greater abundances of genera *Bacillus*, *Lactobacillus*, and *Sphingomonas* was characterized in the patients with a better prognosis [150]. Interestingly, the authors of this study suggest that the ratio of these differentially abundant taxa, labeled as the ‘microbial dysbiosis index’, has a superior predictive ability over the use of certain diversity indexes or names of bacteria to prognosticate [150].

Lastly, the oral microbial profile has also been implicated as a tool for improving therapeutic effectiveness in oral cancer [87,116,118]. Several in vivo studies have been able to demonstrate inhibition of cellular proliferation in cancer cell lines with the use of antibiotics or inhibitors of the effector molecules, such as Nisin, STAT3 inhibitors, and lentivirus (which blocks the CXCL2/CXCR2 signaling axis) [103,106,118]. These molecules can negate the pro-oncogenic influence of oncobacteria, such as P. *gingivalis*, and thus could be useful, at least as an adjunct, to improve the efficacy of checkpoint blockade and other immunotherapeutics in advanced oral cancers [106,116]. The ability of the microbiome to reshape the TME as a means of enhancing the immunotherapeutic sensitivity of tumors is also a potential approach that could improve therapeutic outcomes in the future [150,151]. The discovery of the immunomodulation ability of the microbes, which eliminates tumors by inducing an antigen-specific immunity against them, has paved the way for the development of immunotherapy as we know it today [152]. Along similar lines, there is a need for further studies that explore various avenues of oral microbiota for their potential therapeutic applications. 

## 4. Unanswered Questions About Oral Dysbiosis in Oral Carcinogenesis 

While some of the above-mentioned studies present several pieces of evidence on the link between oral cancer and oral microbiota, one stronger than the other, there is no prospective controlled trial that could solidify the relationship between these two [102,106,114,117]. This, along with the several inherent methodological drawbacks around the analysis of microbes, has left open some questions that are yet to be addressed. 

### 4.1. Causal Versus Casual Association 

One of the most pressing questions regarding the association of oral dysbiosis with oral cancer is whether the qualitative and quantitative change in the microbial community is responsible for (and involved in) the process of carcinogenesis or whether this dysbiosis is merely a byproduct of the carcinogenic mechanisms. Despite the evidence discussed above, there is a presumption that the shift in microbial abundance could be due to the tumor-induced alterations in TME, which disturb the normal flora and are conducive to opportunistic pathogens repopulation [47,88]. This argument is particularly valid considering that most of the clinical studies on ‘dysbiosis-induced oral carcinogenesis’ and their inferences are mostly based on in vivo studies involving cell lines and a few animal models, with barely any human studies, most of which are only observational. On the other hand, considering that oral carcinogenesis is a multi-factorial and multi-step process, one cannot rule out the possibility of the involvement of microbiota in imparting a few contributing molecular changes, if not as a direct etiological agent, in the process of carcinogenesis [147,153,154]. While there are several studies on oral microbiota, discussed in previous sections, which have shown molecular changes that influence the tumor invasiveness and metastatic rate in cancer cell lines under the controlled situation, whether the results of these studies could be replicated in real-life scenarios is yet to be answered [155]. Furthermore, the oral microbiota and microbiome vary considerably between individuals and even within individuals, depending on numerous day-to-day factors, both environmental and personal, which could add to the argument on the reliability of existing clinical studies. 

### 4.2. Role of Confounding Factors 

The oral microbiota in humans is strongly influenced by geographical location, ethnicity, and gender [96,122]. Apart from the site-specific variation in distribution of oral microbiome discussed in earlier sections, the make-up of oral microbiota, particularly of the non-core or ‘variable microbiome’, also changes in an individual from time to time, depending on the age, dietary habits, fasting state, oral hygiene practices, use of alcohol, tobacco, type of alcohol/tobacco, co-morbidities—both local and systemic, medications, radiation exposure, and more [88,89,95,98,122,156,157,158]. During clinical studies, if these variations are not accounted for (some of them are difficult to account for), the reliability of their results could be affected [88,95]. Moreover, the identification and characterization of oral microbiota could also be affected by the procedural variations in the form of the method used for analyzing bacteria (conventional culture method, sanger sequencing, and 16S rRNA metagenomics), the specimen used for analysis (saliva, mucosal surface, or core tissue), the time of the day of specimen collection, the number of specimens collected, the collection procedure (rinse, passive wash, swab, scrapping or biopsy) the pre-collection steps (use of oral washes, type of reagent used), and the selection of controls (normal healthy controls or contralateral normal mucosal lesions from cancer patients) [89,96,121,122,159]. This methodological variation(s) could explain some of the drastic inconsistencies found in the literature with regard to reported profiles of microbiota in supposedly identical disease states. Furthermore, studies have also used different sequencing regions (between V4-V5, V4, V3-V5, V1-V3) as targets to identify microbiomes, which could have also had some bearing on their outcomes [47,84,90,91,99]. 

### 4.3. Future Direction

While the causality and dose–response relationship may not have been clearly established between oral dysbiosis and oral carcinogenesis in a real-world scenario, there is a considerable body of emerging evidence to support a plausible causal relationship between them. A few animal models, such as the one described by Binder Gallimid, Ren, Harrandah, and Li, and the uncovering of a few effective pathways, such as the production of acetaldehyde, alteration of TME, inhibition of NK cells, etc., show that this association is likely to be more than merely casual [102,106,111,114,117,129,134]. However, further high-quality, prospective studies, both controlled trials and real-world studies, with large sample sizes and other methodological accuracies to maintain adequate statistical power, are needed in the future to establish the role of oral microbiota in oral carcinogenesis. Particularly, large population-based cohort studies that measure the baseline microbial profiles of a defined population and longitudinally follow up the participants with serial microbial assessments until a meaningful endpoint, could provide a clear answer [88,122]. The measurement of the microbiome in future studies needs to be standardized and reproducible, with more precise measurement conditions and accurate assessment methods [122]. Despite the obvious challenges, to improve our knowledge on the participation of the oral microbiota in the development of oral cancer, it is necessary to to study the oral microbiota (through a standardized method of sample collection) longitudinally, in both healthy patients as well as high-risk patients, through the various phases of transition from OPMD to oral carcinogenesis, and after treatment. The utilization of artificial intelligence and deep learning techniques could aid researchers to explore the causal link between dysbiotic profile(s) and carcinogenesis much more proficiently, quickly and with more reliability. Furthermore, exploring the holistic interactions between the microbes and hosts, developing new conceptual frameworks, interdisciplinary collaborations, and establishing other microbiome databases in the model of HOMD are some of the other priorities that could advance the field of human microbiome research [160]. These understandings would also pave the way to explore the preventive (as well as therapeutic) role of probiotics and other related therapies in halting the transition of OPMD into oral cancer via the restoration of the oral microbiome. 

## 5. Conclusions

While the current literature may not provide an accurate answer to whether the peculiar dysbiotic profile is an etiological factor for oral cancer, it provides unfathomable evidence on the nexus between oral dysbiosis and oral cancer and also points towards a possible therapeutic avenue in the microbiome to reduce the proliferation of cancer cells. Further studies are required to establish a direct link between oral dysbiosis and oral carcinogenesis. 

## Data Availability

Not applicable.

## Abbreviations

The following abbreviations are used in this manuscript:

ABS	Auto-brewery syndrome
ADH	Alcohol dehydrogenase
ALDH	Acetaldehyde dehydrogenase
CDH1	Cadherin-1
CEACAM	Carcinoembryonic antigen-related cell adhesion molecule 1
COX	Cyclooxygenases
CXCL2	Chemokine (C-X-C motif) ligand 2
CXCR2	C-X-C motif chemokine receptor 2
DC	Dendritic celss
DNA	Deoxyribonucleic acid
EBV	Epstein Barr virus
EMT	Epithelial mesenchymal transition
FAK	Focal Adhesion Kinase
GLUT1	Glucose transporter 1
G2/M	Gap2/Mitosis
HIV	Human immunodeficiency virus
HMP	Human microbiome project
HOMD	Linear dichroism
HPV	Human papillomavirus
IL	Interleukin
JAK1	Janus kinase 1
miR	MicroRNA
MMP	Matrix metalloproteinases
NF-κB	Nuclear Factor-Kappa B
NK	Natural killer cells
OPMD	Oral potentially malignant disorder
PDCD4	Programmed cell death factor 4
PGE_2_	Prostaglandin E_2_
PPL	Periodontal pathogen—low
PPH	Periodontal pathogen—high
RNA	Ribonucleic acid
STAT3	Signal transducer and activator of transcription 3
TGF-β	Transforming growth factor beta
TIGIT	T cell immunoglobulin and ITIM domain
TME	Tumor microenvironment
TNFα	Tumor necrosis factor alpha
TLR	Toll-like receptor
ZEB1	Zinc finger E-box-binding homeobox 1
